# The 26S Proteasome Degrades the Soluble but Not the Fibrillar Form of the Yeast Prion Ure2p *In Vitro*

**DOI:** 10.1371/journal.pone.0131789

**Published:** 2015-06-26

**Authors:** Kai Wang, Virginie Redeker, Karine Madiona, Ronald Melki, Mehdi Kabani

**Affiliations:** Paris-Saclay Institute of Neuroscience, Centre National de la Recherche Scientifique, Gif-sur-Yvette, France; Deutsches Zentrum für Neurodegenerative Erkrankungen e.V., GERMANY

## Abstract

Yeast prions are self-perpetuating protein aggregates that cause heritable and transmissible phenotypic traits. Among these, [*PSI* ^+^] and [*URE3*] stand out as the most studied yeast prions, and result from the self-assembly of the translation terminator Sup35p and the nitrogen catabolism regulator Ure2p, respectively, into insoluble fibrillar aggregates. Protein quality control systems are well known to govern the formation, propagation and transmission of these prions. However, little is known about the implication of the cellular proteolytic machineries in their turnover. We previously showed that the 26S proteasome degrades both the soluble and fibrillar forms of Sup35p and affects [*PSI* ^+^] propagation. Here, we show that soluble native Ure2p is degraded by the proteasome in an ubiquitin-independent manner. Proteasomal degradation of Ure2p yields amyloidogenic N-terminal peptides and a C-terminal resistant fragment. In contrast to Sup35p, fibrillar Ure2p resists proteasomal degradation. Thus, structural variability within prions may dictate their ability to be degraded by the cellular proteolytic systems.

## Introduction

Yeast prions are self-perpetuating protein aggregates that manifest as non-Mendelian and cytoplasmically inherited dominant phenotypic traits. [*PSI* ^+^] and [*URE3*] stand out as the best known and the most documented yeast prions, and result from the aggregation of the soluble forms of Sup35p, a translation termination factor, and Ure2p, a negative regulator of nitrogen catabolism, respectively, into infectious entities (reviewed in [1, 2]).

Purified Sup35p and Ure2p are both able to spontaneously assemble into protein fibrils under physiological conditions [3–6]. These fibrillar assemblies are infectious in that they efficiently induce the prion state when re-introduced into prion-free yeast cells [7–11]. Assembly of Sup35p and Ure2p into fibrils strictly depends on the presence of a glutamine and asparagine-rich domain, referred to as the prion domain (PrD), and located at the N-terminus of both proteins [1]. Furthermore, Sup35p and Ure2p can populate an ensemble of structurally different and heritable molecular conformations that lead to a wide range of phenotypically distinct [*PSI* ^+^] and [*URE3*] prion strains, respectively [7, 8, 11–18]. However, the fibrillar assemblies formed by Sup35p and Ure2p in our comparable experimental conditions are structurally unrelated. Sup35p fibrils are believed to have an amyloid structure, with the PrD of individual Sup35p monomers stacked along the fibrils axis and the globular C-terminal domains protruding from the fibrils core [14, 19–23], while both the N- and C-terminal moieties are integral parts of Ure2p fibrils under our experimental conditions [8, 24–29]. As PrDs in isolation assemble into prion-inducing fibrils, they were often used as proxies to decipher the structure of the full-length prion assemblies ([1] and references therein). Nonetheless, we showed for both Sup35p and Ure2p that the latter assemblies are structurally and functionally different from those formed by the full-length proteins [8, 9, 19–21, 28–30].

*De novo* formation, propagation and elimination of [*PSI* ^+^] and [*URE3*], as well as that of other yeast prions, is highly dependent on the interplay between actors of the cellular protein folding and quality control machineries, among which the Hsp70, Hsp40 and Hsp104 molecular chaperones [2, 31]. However, very little is known on the potential role of the major cellular proteolytic systems, the ubiquitin-proteasome system (UPS) and autophagy, on the turnover of the soluble, oligomeric or fibrillar intermediates populated by yeast prions [32]. Links between the UPS and [*PSI* ^+^] were previously reported but did not directly address whether the proteasome degrades prion particles [33, 34]. We recently demonstrated that reducing intracellular proteasome pools caused Sup35p accumulation and defects in [*PSI* ^+^] formation and propagation [32]. We showed that purified yeast 26S proteasome was able to degrade Sup35p *in vitro*, both in its soluble native form and in its highly ordered fibrillar form associated with [*PSI* ^+^] [32]. These findings revealed a possible and overlooked role of the proteasome in the turnover of prion assemblies [32].

Here, we show that the turnover of Ure2p *in vivo* is dependent on proteasomal activity. We assess whether the soluble and fibrillar forms of Ure2p are substrates of the 26S proteasome *in vitro*. We show that soluble Ure2p is readily degraded by the 26S proteasome in an ubiquitin-independent manner. As observed previously for Sup35p, the degradation of soluble Ure2p proceeds from the N-terminal end yielding an array of amyloidogenic peptides. Furthermore, we show that a deletion of residues 3 to 25 completely abolishes degradation, suggesting this region bears an important degron required by the proteasome to engage Ure2p. Remarkably and contrary to what we observed for Sup35p amyloid fibrils, we show that native-like Ure2p fibrils resist proteasomal degradation.

## Results

### Soluble native Ure2p is a proteasome substrate *in vivo* and *in vitro*

**Fig 1:**
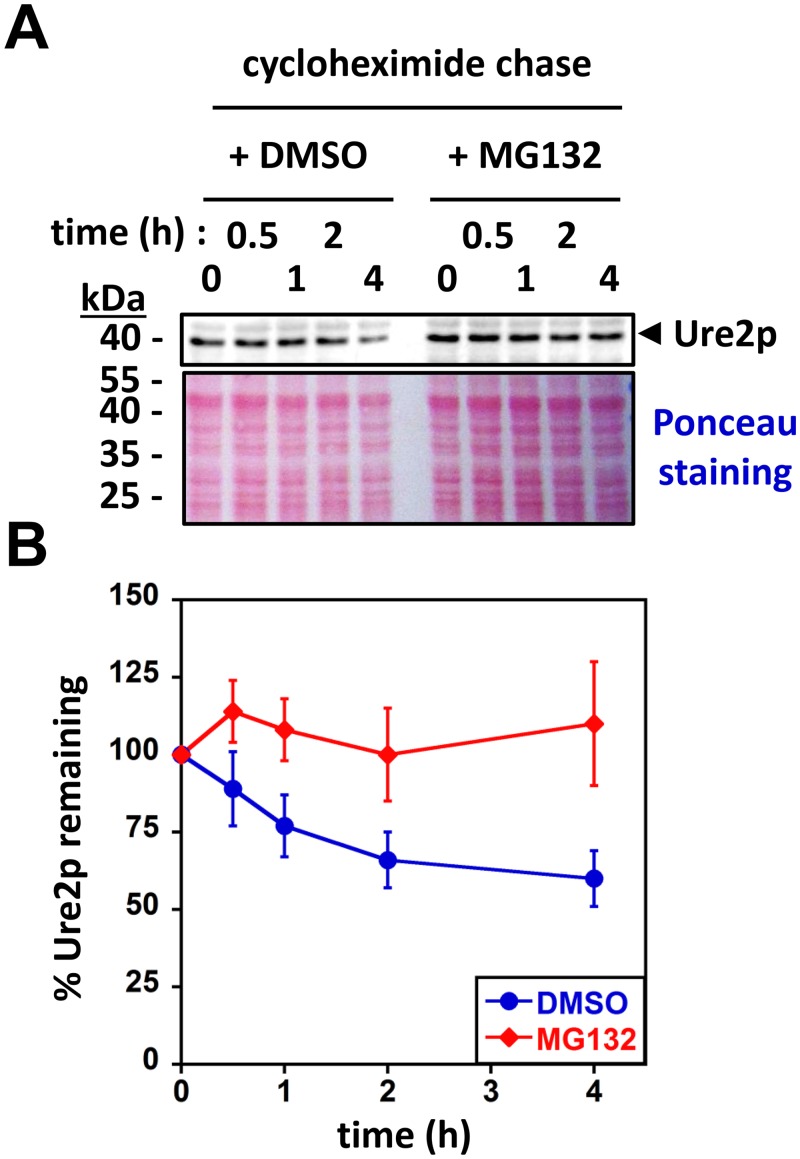
Soluble Ure2p is a proteasomal substrate *in vivo*. **(A)** Yeast cells overexpressing Ure2p (see Materials and Methods) were grown to mid-log phase and then treated with DMSO or MG132 (50 μM) for 30 min at 30°C. Protein expression was shut off by the addition of cycloheximide (100 μg.ml^-1^), and aliquots were withdrawn at the times indicated (in hours). Cell extracts were prepared and analyzed by SDS-PAGE followed by Western blotting using anti-Ure2p antibodies (upper panel) or Ponceau staining (lower panel). **(B)** Quantification of western blots such as those shown in (A) was performed using ImageJ. The amount of Ure2p at time zero was set to 100% (data points represent the mean ± SE of independent experiments performed in triplicate).

To determine whether Ure2p is a proteasomal substrate *in vivo*, we assessed its stability in the presence or absence of proteasome inhibitors using cycloheximide-chase experiments [35]. In order to allow the detection of Ure2p in cell extracts by Western blot, these experiments were carried out in a prion-free [*ure-0*] wild-type yeast strain bearing a plasmid that drives *URE2* overexpression under the control of a tet-inducible promoter (see Materials and Methods) [36]. As described previously [37], we found that Ure2p is slowly degraded over time (Fig 1A and 1B). In contrast with what we observed with DMSO, Ure2p degradation was abolished for over 4h in the presence of the proteasome inhibitor MG132 (Fig 1A and 1B). This suggests that the proteasome contributes to soluble Ure2p degradation. We were not able to make similar observations in cells harboring the [*URE3*] phenotype. Nonetheless, the experiments depicted in Fig 1A and 1B prompted us to investigate the proteasomal degradation of Ure2p in well-defined *in vitro* assays.

**Fig 2:**
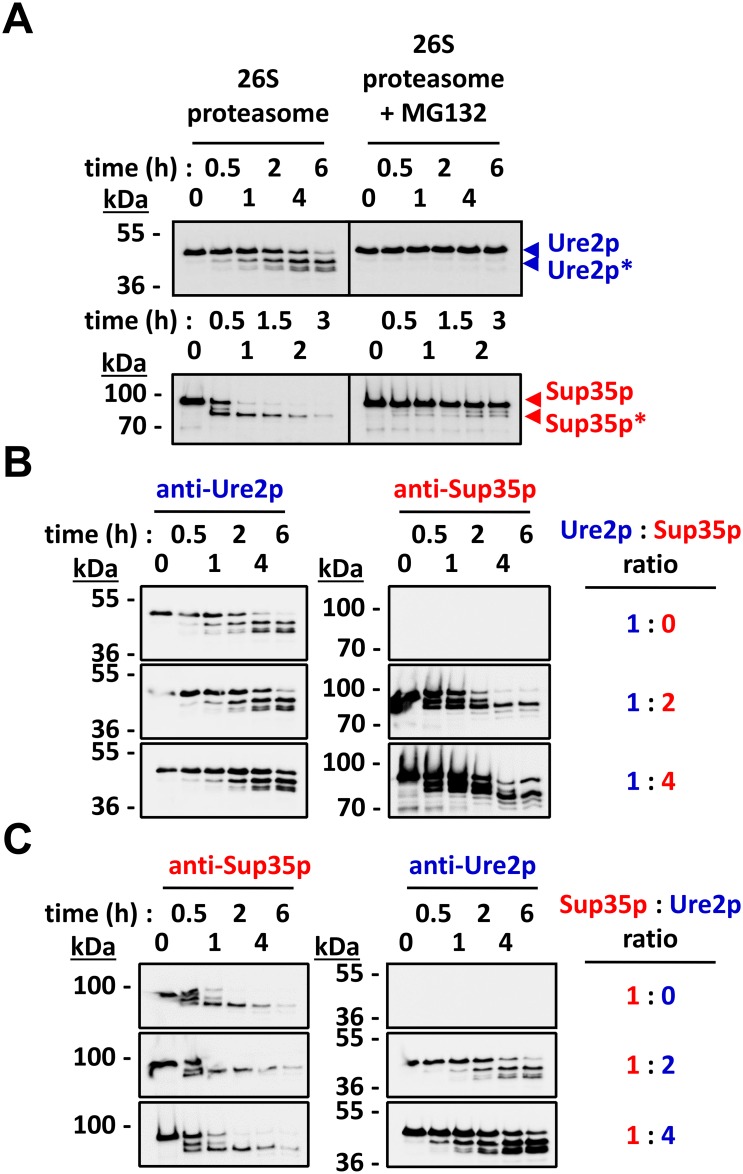
Soluble Ure2p is a proteasomal substrate *in vitro*. **(A)** Purified 26S proteasomes (2 nM) were mixed with purified soluble Ure2p (250 nM) (upper panel) or Sup35p (125 nM) (lower panel) in the presence of 2.5 mM ATP, with or without MG132 (100 μM), as indicated. The reaction mixes were incubated at 30°C under mild agitation (<300 rpm). At the indicated time points, aliquots were removed and analyzed by SDS-PAGE and Western blotting using anti-Ure2p or anti-Sup35p antibodies. Ure2p* and Sup35p* indicate proteasome-resistant fragments. **(B)** Purified 26S proteasomes (2 nM) were mixed with purified Ure2p (250 nM) and without or with increasing concentrations of purified Sup35p (250 nM to 1 μM), in the presence of 2.5 mM ATP. Reaction mixes were incubated and analyzed as described in (A). **(C)** Purified 26S proteasomes (2 nM) were mixed with purified Sup35p (125 nM) and without or with increasing concentrations of purified Ure2p (250 nM to 500 nM), in the presence of 2.5 mM ATP. The reaction mixes were incubated at 30°C under mild agitation (<300 rpm). Reaction mixes were incubated and analyzed as described in (A).

We previously showed that purified yeast 26S proteasomes were able to degrade soluble native Sup35p *in vitro* [32]. Proteasomal degradation of Sup35p proceeded sequentially from the N-terminal PrD, generating an array of overlapping amyloidogenic peptides and a C-terminal proteasome-resistant Sup35p fragment spanning residues 83–685 [32]. To determine whether Ure2p is degraded by yeast 26S proteasomes *in vitro* and compare its proteolytic processing to that of Sup35p, we incubated soluble Ure2p in the presence of purified yeast 26S proteasomes and ATP and in the presence or absence of the proteasome inhibitor MG132 at 30°C. Aliquots were withdrawn at time intervals and analyzed by SDS-PAGE and Western blot using anti-Ure2p antibodies. A control reaction where Sup35p was incubated under exactly the same conditions was run in parallel. Fig 2A (upper panel, left) shows that full-length Ure2p is degraded in a time-dependent manner by the 26S proteasome. The proteasome inhibitor MG132 abolished degradation (Fig 2A, upper panel, right). Beside a major proteolytic fragment, denoted Ure2p*, two lower molecular-weight Ure2p fragments persisted several hours after the onset of the reaction (Fig 2A, upper panel, left). Overall, Ure2p degradation is reminiscent of what we observe for Sup35p in the control reaction (Fig 2A, lower panels and as we previously reported, [32]).

We next compared the binding and degradation efficiency of Ure2p and Sup35p by yeast 26S proteasomes. A constant amount of yeast 26S proteasomes and Ure2p was incubated in the absence or presence of increasing amounts of Sup35p. The reaction mixtures were incubated at 30°C and aliquots were withdrawn at the indicated time and analyzed by SDS-PAGE and western blot using anti-Ure2p and anti-Sup35p antibodies. Fig 2B shows that both Ure2p and Sup35p were degraded under all conditions. The degradation of Ure2p was progressively slowed, but not abolished, with the highest amounts of Sup35p in the reaction mixture (Fig 2B, bottom panels). Both Ure2p and Sup35p were degraded by the proteasome in the reciprocal experiment where a constant amount of yeast 26S proteasomes and Sup35p was incubated in the absence or presence of increasing amounts of Ure2p (Fig 2C). However, in this case, the degradation of Sup35p was not significantly slowed by the addition of up to a four-fold molar excess of Ure2p (Fig 2C, bottom panels). This suggests that soluble Sup35p is a preferred proteasome substrate compared to soluble Ure2p, at least in these experimental conditions.

### Proteasomal degradation of Ure2p generates amyloidogenic peptides from the N-terminal moiety

**Fig 3:**
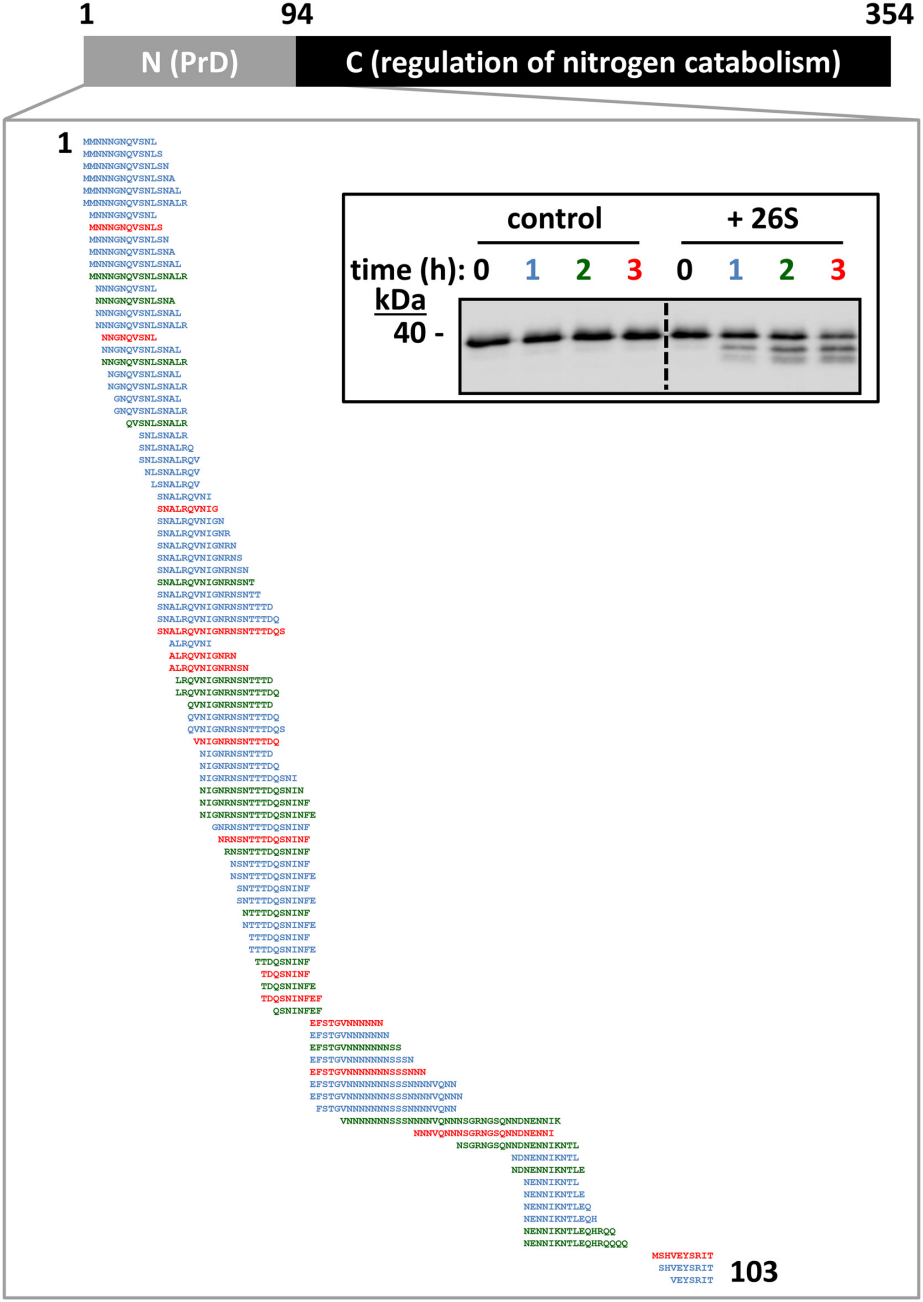
Ure2p degradation by the 26S proteasome yields ~10–30 aminoacids-long peptides originating from the N-terminal end of the protein. Purified soluble Ure2p (1 μg) was incubated without or with purified 26S proteasome (0.4 μg) in the presence of 2.5 mM ATP at 30°C under mild agitation (<300 rpm) for 0, 1, 2 or 3 h. Peptides produced during the incubation were identified by nanoLC-LTQ-Orbitrap mass spectrometry (see also S1 Table). These peptides were not produced when Ure2p was incubated alone (data not shown). The color code reflects the time point at which each individual peptide was first detected, as indicated. **(Inset)** Aliquots from the reaction mixes were analyzed by SDS-PAGE and Western blot using anti-Ure2p antibodies.

We next identified the proteolytic products generated upon soluble Ure2p degradation by the 26S proteasome. Ure2p was incubated alone or in the presence of 26S proteasomes for up to 3 h at 30°C, and the reaction mixtures analyzed by western blot (Fig 3, inset) and nanoLC-LTQ-Orbitrap mass spectrometry. The degradation of Ure2p by the 26S proteasome generated 94 different ~10- to 30-amino-acid-long peptides, spanning the first 103 residues of the protein (Fig 3, S1 Table). None of these peptides was identified in the control reactions without 26S proteasomes, indicating they correspond to specific products of proteasome-dependent Ure2p proteolysis. Most peptides were generated within 1 h of incubation, while others were detected after 2 h or 3 h of incubation (Fig 3, S1 Table). This time-dependent mass-spectrometry analysis suggests that Ure2p degradation by the proteasome proceeds sequentially from the N-terminal end of the protein towards the C-terminus (Fig 3, S1 Table). No peptides spanning residues 103 to 354 were identified in our mass spectrometry analysis within the time frame of the experiment. The proteasomal degradation pattern of Ure2p we observed (Fig 2A, upper panel left and Fig 3, inset) is thus due to processive cleavage of Ure2p N-terminal PrD. The compactly folded globular C-terminal domain of Ure2p [38] appeared to resist proteasomal degradation. Thus, the proteasomal degradation of soluble Ure2p *in vitro* resembles that of Sup35p [32]. Indeed, both proteins are sequentially degraded via their intrinsically disordered N-terminal PrDs, yielding amyloidogenic peptides and proteasome-resistant fragments encompassing their folded and functional C-terminal domains (Figs 2and 3) [32].

### Deletion of residues 3–25 prevents proteasomal degradation of soluble Ure2p

**Fig 4:**
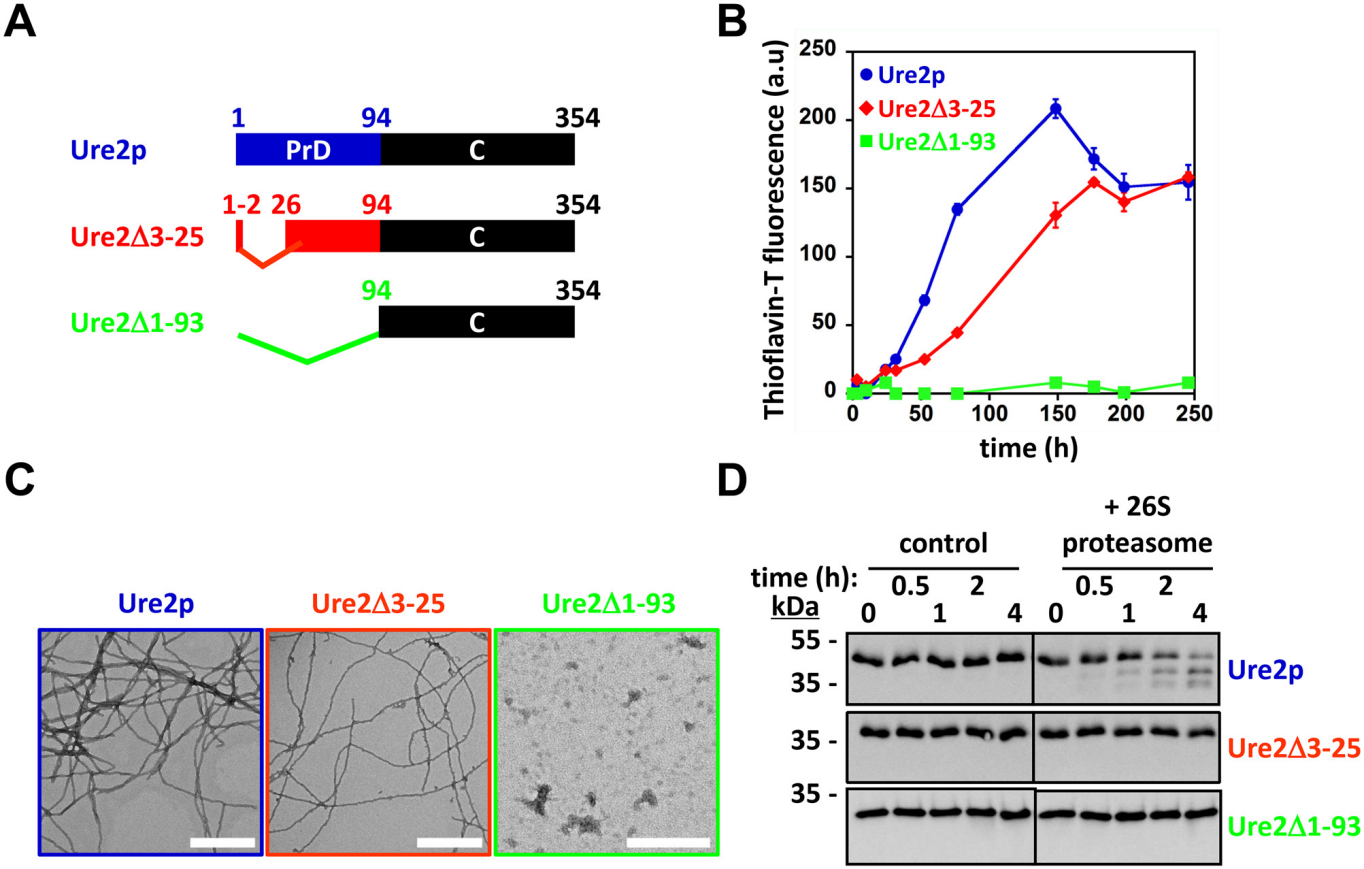
The deletion of residues 3–25 prevents the proteasomal degradation of Ure2p. **(A)** Cartoon representation of the Ure2p variants used in this study. **(B)** Time-courses of Ure2p, Ure2Δ3–25 and Ure2Δ1–93 (25 μM) assembly at 6°C, monitored by thioflavin T binding (a.u., arbitrary units). Data represent the mean of three independent experiments ± SE **(C)** Negative-stained electron micrographs of Ure2p, Ure2Δ3–25 and Ure2Δ1–93 (25 μM) assemblies after 30 days of incubation at 6°C (scale bar: 500 nm). Only amorphous aggregates were detected for Ure2Δ1–93. **(D)** Purified Ure2p, Ure2Δ3–25 or Ure2Δ1–93 (250 nM) were incubated with or without purified 26S proteasomes (2 nM), as indicated, and in the presence of 2.5 mM ATP. The reaction mixes were incubated at 30°C under mild agitation (<300 rpm). At the indicated time points, aliquots were removed from the reaction mix and analyzed by SDS-PAGE and Western blotting using anti- Ure2p antibodies.

We previously showed that a truncated form of Sup35p lacking the N-terminal 82 amino acid residues (Sup35Δ1–82) resists proteasomal degradation suggesting a prominent role for Sup35p PrD in recognition and degradation by the 26S proteasome [32]. The results presented in Figs 2 and 3 suggest that Ure2p PrD behaves as a degron that is recognized and engaged by the 26S proteasome. This is supported by the finding that a truncated form of Ure2p lacking its N-terminal domain (Ure2Δ1–93, Fig 4A), used to solve the structure of the compactly folded domain of Ure2p [39], and that is unable to assemble into fibrils (Fig 4B and 4C), fully resists proteasomal degradation (Fig 4D). It should be noted here that a Ure2p variant lacking its prion domain (Ure2Δ2–94) was shown to be unstable *in vivo* [37]. Nor the reasons behind this instability, nor the protease(s) responsible for Ure2Δ2–94 degradation *in vivo* were identified [37]. Therefore, Ure2Δ1–93 (or UreΔ2–94) may very well be highly resistant to proteasomal degradation, as shown in Fig 4D, but not to other proteolytic machineries.

To further narrow down the stretch of Ure2p amino acid residues specifically recognized and engaged by the proteasome, we generated a number of deletions within the N-terminal domain of Ure2p and assessed the resistance of the truncated Ure2p to proteasomal degradation. The Ure2p variant bearing the shortest deletion, Ure2Δ3–25 (Fig 4A), that retains assembly propensity (Fig 4B and 4C) fully resisted proteasomal degradation (Fig 4D). This observation suggests that the degron within Ure2p spans at most residues 3–25. This is consistent with previous observations we made indicating that the first 25 residues of soluble Ure2p fall within a region of the PrD readily accessible to proteolytic cleavage and hydrogen/deuterium exchange [25, 40].

### Fibrillar Ure2p is not degraded by the proteasome *in vitro*

**Fig 5:**
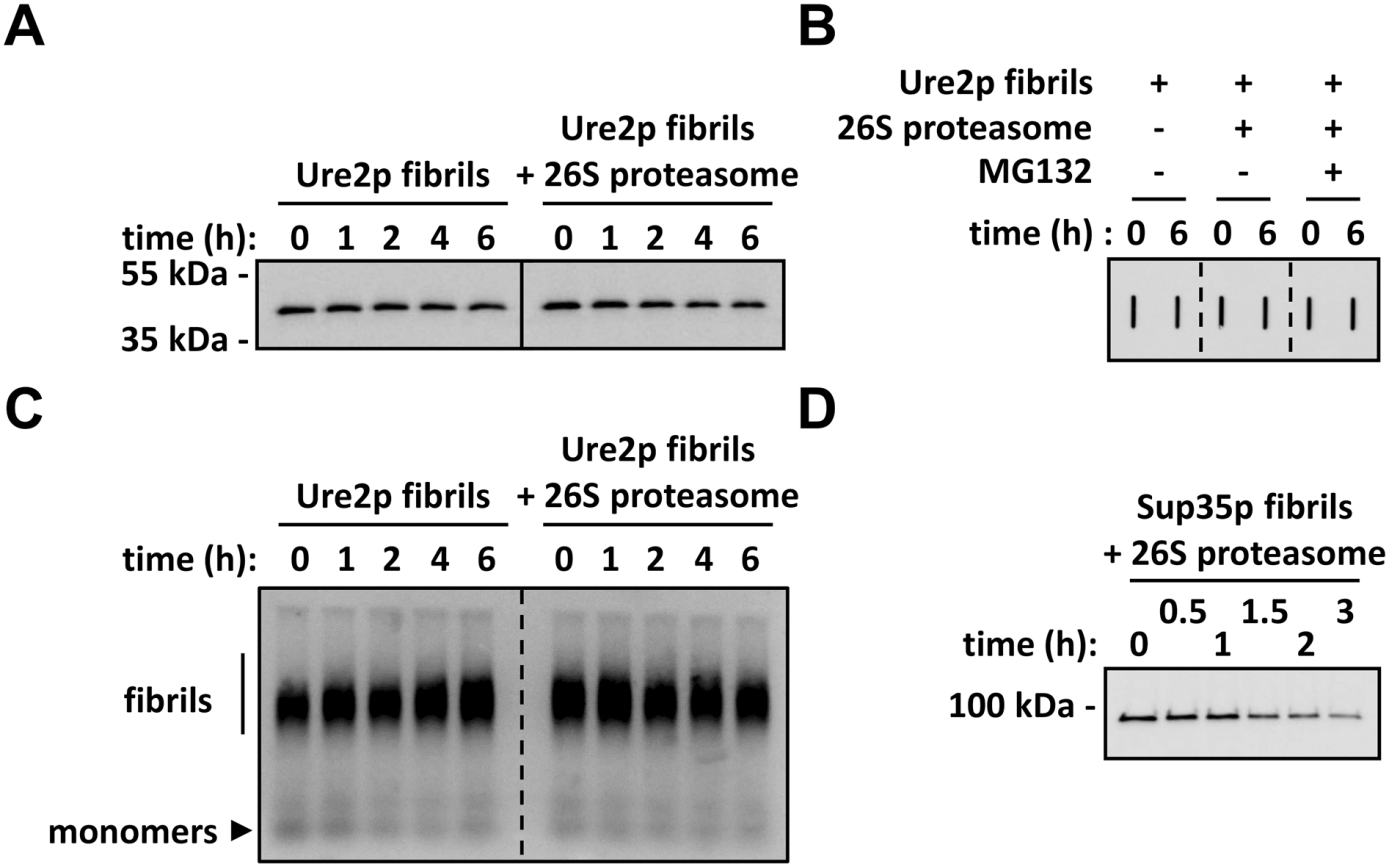
Fibrillar Ure2p is not degraded by the 26S proteasome. **(A)** Ure2p fibrils (2 μg) were incubated at 30°C under mild agitation (<300 rpm) in the presence of 2.5 mM ATP, with or without 26S proteasomes (1.6 μg), as indicated. Aliquots were removed at time intervals and analyzed by SDS-PAGE followed by Western blotting using anti-Ure2p antibodies. **(B)** Ure2p fibrils were incubated with or without 26S proteasomes and MG132 (100 μM), as indicated in (A). At the indicated time points, aliquots were diluted four-fold in proteasome assay buffer and then filtered through a cellulose acetate membrane (0.2 μm pore size) using a slot-blot vacuum manifold. Each well was then washed twice with 200 μL assay buffer and the membranes were immunostained with anti-Ure2p antibodies. **(C)** Ure2p fibrils were incubated with or without 26S proteasomes, as indicated in (A). Aliquots were withdrawn at time intervals and analyzed by SDD-AGE followed by immunoblotting with anti-Ure2p antibodies **(D)** Sup35p fibrils (1 μg) were mixed with purified 26S proteasomes (0.4 μg) in the presence of 2.5 mM ATP. The reactions mixes were incubated at 30°C under mild agitation (<300 rpm), and at the indicated time points, aliquots were removed and analyzed by SDS-PAGE followed by Western blotting using anti-Sup35p antibodies.

We previously demonstrated that the 26S proteasome degrades Sup35p fibrils *in vitro*, thereby abolishing their infectivity in protein transformation experiments [32]. To determine whether Ure2p fibrils are degraded by the proteasome, Ure2p fibrils were incubated at 30°C with or without 26S proteasomes in the presence of ATP under mild agitation and aliquots, withdrawn at the indicated time, were immunoblotted using anti-Ure2p antibodies after SDS-PAGE or trapping on cellulose acetate membranes [41]. The results presented in Fig 5A and 5B, clearly indicate that Ure2p fibrils were not degraded by the 26S proteasomes under our experimental conditions as the intensity of Ure2p band on the SDS-PAGE and the filter trap remained unchanged upon incubation for 6h in the presence of 26S proteasomes. To strengthen this observation and rule out possible proteasome-mediated fibrils remodeling, the integrity of preformed fibrils incubated for up to 6h with the 26S proteasomes and ATP at 30°C was assessed by SDD-AGE [32, 42]. Fig 5C shows that the size distribution of Ure2p fibrils is unaffected by their incubation with the 26S proteasome, and that the intensity of the band remained unchanged. We conclude from these observations that the 26S proteasome neither remodels nor degrades preformed Ure2p fibrils. A control reaction ran in parallel, shows that preformed Sup35p fibrils are degraded by the 26S proteasome (Fig 5D), as described previously [32].

## Discussion

Yeast prions have been proposed to act as protein-only epigenetic elements which increase phenotypic diversity by altering gene expression, thus allowing yeasts to rapidly and transiently adapt to changes in the surrounding environment [1, 31]. Thus, when considering a population of yeast cells, harboring prions can be deemed beneficial as it allows the survival of a subset of individuals from that population in challenging growth conditions. Nonetheless, for individual yeast cells, prions constitute an abnormal situation-akin to degenerative conformational disorders- that needs to be dealt with [9, 31, 43–46]. An arsenal of molecular chaperones controls prion formation and faithful propagation, yet little is known about the proteolytic clearance of the soluble, oligomeric or fibrillar molecular species populated by these prions, specifically by the UPS or autophagy [1, 2, 31]. We previously uncovered a role for the proteasome in the life cycle of [*PSI* ^+^] and demonstrated that the proteasome has the intrinsic ability to degrade the soluble and fibrillar forms of Sup35p [32].

Whether this is a generic property allowing the proteasome to modulate the propagation of other prions or not is an open question. Indeed, yeast cells host prion proteins with unrelated primary structures and cellular functions [1, 2, 31]. Therefore both similar and divergent features between different prions were described at the cellular (*e*.*g*. mechanisms of *de novo* formation and dependence on molecular chaperones) and molecular (*e*.*g*. biochemical and structural characteristics of prion high molecular weight assemblies) levels [1, 2, 31]. Thus, the turnover of the various molecular entities populated by different yeast prions may similarly depend upon different mechanisms and cellular factors.

The cellular turnover of Ure2p (Fig 1A and 1B) and Sup35p [32] both depend on the proteasome. As the similarities and differences between Sup35p and Ure2p are well documented, we compared their degradation by the 26S proteasome in a well-defined *in vitro* system. The proteasomal degradation of soluble Sup35p and Ure2p is similar (Fig 2): *i)* their unstructured N-terminal PrDs serve as a molecular tether allowing the proteasome to bind and to sequentially degrade both proteins, and deletion of the PrD results in complete protection against proteasomal action (compare Fig 4 to Fig 4 in [32]); *ii)* the degradation of Ure2p and Sup35p PrDs yields an array of overlapping ~10-30-amino-acid-long peptides with predicted amyloidogenic propensity (compare Fig 3 to Fig 5 in [32]); *iii)* their compactly folded functional domains resist proteasomal degradation (Figs 2 and 4). Interestingly, at least in our experimental conditions, the proteasomal degradation of Sup35p and Ure2p did not require ubiquitination [32]. It should be noted that while we and others could not detect ubiquitinated Sup35p species *in vivo* [32–34, 47], Ure2p was fished out in a proteomic screen for ubiquitinated proteins in yeast [48]. Thus, while ubiquitination may modulate proteasomal degradation of yeast prions *in vivo*, the presence of an unstructured domain, a common feature of most if not all prions, appears, necessary and sufficient to trigger proteasomal degradation [32, 49]. The minimal requirements needed for these unstructured domains to serve as proteasomal degrons remain to be determined, bearing in mind that the flexibility of the PrDs *in vitro* and *in vivo* following interaction with their cognate compactly folded functional domain and/or other cellular partners, may not compare.

Another important observation we made is that while Sup35p fibrils are degraded by the proteasome (Fig 5D) [32], Ure2p fibrils are not (Fig 5A, 5B and 5C). No fully proteasome-resistant fragment is formed upon degradation of Sup35p fibrils, contrary to that of soluble Sup35p [32]. We previously attributed this either to major conformational changes occurring in Sup35p upon assembly and that would be retained upon spontaneous dissociation of Sup35p monomers from the fibril ends, or to the exposure of Sup35p degron to the solvent in the fibrillar form [32]. The finding that Ure2p fully resists degradation in its fibrillar form implies that its degron is inaccessible within the fibrils in agreement with the observation that Ure2p N-terminal domain has decreased solvent accessibility and susceptibility to protease cleavage [25, 40]. Alternatively, dissociation of monomers from fibril ends-which would render them accessible to the proteasome- may not occur for Ure2p, at least *in vitro*. In agreement with the latter hypothesis is the finding that Ure2p fibrils are more resistant to SDS treatment than Sup35p fibrils (unpublished observations).

The structural variability within prion assemblies in a cellular context is expected to dictate their interaction with proteolytic machineries in general and the proteasome in particular. It will be important for future studies to isolate these species directly from cells and document their biophysical and structural characteristics to gain better insight into the proteolysis of prions.

## Materials and Methods

### Proteins

Recombinant untagged Ure2p, Ure2Δ3–25, Ure2Δ1–93 and hexa-histidine tagged Sup35p were overexpressed in *E*. *coli* strain BL21-CodonPlus and purified according to previously published procedures [4, 5, 39]. The assembly of Sup35p and Ure2p into protein fibrils was performed at 6°C under very mild agitation (<100 rpm) in Assembly Buffer 1 (50 mM Tris-HCl, pH 8.0, 200 mM NaCl, 5% glycerol, 5 mM β-mercaptoethanol, 10 mM MgCl_2_, 2 mM EGTA) and Assembly Buffer 2 (20 mM Tris.HCl pH 7.5, 200 mM KCl, 1mM EGTA, 1 mM DTT), respectively, as described previously [4, 5, 32, 39]. Assembly reactions were monitored by thioflavin-T binding [4, 25]. Yeast 26S proteasomes were purified by affinity chromatography as described before [32, 50]. The integrity and activity of the 26S proteasome preparations was assessed as described previously [32].

### Cycloheximide-chase experiments

The *URE2* gene was cloned in the pCM252 plasmid [36] to allow its overexpression under the control of a doxycylin-inducible promotor. The resulting pCM252-*URE2* plasmid was then transformed into the BY251 [*ure-0*] wild-type yeast strain [7]. Cycloheximide-chase experiments were performed as described in [35]. Briefly, cells were grown to mid-log phase in minimal medium containing 0.1% proline as the sole nitrogen source. Cells were adjusted to an OD_600nm_~0.5 into fresh medium containing 0.003% SDS and 10 μg.ml^-1^ doxycylin. After 3h of incubation at 30°C under agitation, MG132 (50 μM) or the control buffer DMSO were added. After 30 min of incubation, cycloheximide was added at a final concentration of 100 μg.ml^-1^. Aliquots were taken at time intervals, cells were harvested by centrifugation and snap-frozen in liquid nitrogen. Cell extracts were prepared as described previously [32] and analyzed by western blotting using anti-Ure2p antibodies.

### Proteasome degradation assays *in vitro*

Proteasome degradation assays *in vitro* were performed in proteasome assay buffer (50 mM Tris.Cl pH 7.5, 50 mM KCl, 10 mM MgCl_2_, 1 mM DTT, 0.1% Tween-20, 2.5 mM ATP, 10% glycerol) essentially as described before [32]. Briefly, the indicated amounts of purified 26S proteasomes and substrates (soluble or fibrillar Ure2p or Sup35p) were mixed in proteasome assay buffer and incubated at 30°C under mild agitation (<300 rpm). When indicated, MG132 was present at a final concentration of 100 μM. Aliquots were removed at time intervals and analyzed by SDS-PAGE, semi-denaturing detergent gel electrophoresis (SDD-AGE) or filter-trap. For SDS-PAGE, aliquots were mixed with an equal volume of 2X urea sample buffer (125 mM Tris·Cl pH 6.8, 8 M urea, 4% SDS, 20% glycerol, 10% β-mercaptoethanol, 0.05% bromophenol blue) [32] and heated at 90°C for 5 min. For SDD-AGE analysis, aliquots were mixed with an equal volume of 2X SDD-AGE sample buffer (1X Tris-Acetate-EDTA (TAE), 10% glycerol, 4% SDS, 0.025% bromophenol blue), incubated for 15 min at room temperature, and then separated on 1.5% agarose gels in 1X TAE buffer containing 0.1% SDS. Proteins were then blotted onto nitrocellulose membranes by capillary transfer in Tris-buffered saline (TBS). For filter-trap assays, reactions were diluted four-fold in proteasome assay buffer and then filtered through cellulose acetate membranes (0.2 μm pore size) using a slot-blot vacuum manifold. Membrane wells were then washed twice with 200 μL proteasome assay buffer. In all cases, membranes were analyzed by Western blot using anti-Sup35p or anti-Ure2p antibodies.

### Electron microscopy

Samples were stained with 1% uranyl acetate on carbon-coated grids and imaged in a Jeol 1400 transmission electron microscope. Images were recorded with a Gatan Orius CCD camera (Gatan, Pleasanton, CA) and processed with the ImageJ software (NIH).

### Identification of Ure2p degradation products by mass spectrometry

Purified Ure2p (1 μg) was incubated with or without 26S proteasome (0.4 μg) in proteasome assay buffer at 30°C under mild agitation (<300 rpm) for 1, 2 or 3 hours. To identify the peptides generated at each time point, 80 μL of each reaction were cleared by centrifugation at 15000 x g for 10 min, desalted and concentrated on a ZIP-TIP C18 (Millipore), and then eluted in 5 μL 20% acetonitrile, 0.1% trifluoroacetic acid. The final volume was brought to 20 μL with 0.1% trifluoroacetic acid. Peptides (10 μL) were separated and identified by nanoLC-LTQ-Orbitrap mass spectrometry as described previously [32].

## Supporting Information

S1 Table List of peptides generated upon soluble Ure2p degradation by the 26S proteasome and identified by nanoLC-LTQ-Orbitrap mass spectrometry. The position and sequence of each peptide within the primary sequence of Ure2p are indicated, as well as the mass to charge ratio of the peptide that has been fragmented during the nanoLC-MS/MS analysis, the experimental mass (M exp), the theoretical mass (M theor), the mass deviation between the experimental and the theoretical mass (ΔM in ppm) and the mascot ion score for the MS/MS match (score).
(PDF)
