# Supplementary material for: The 26S Proteasome Degrades the Soluble but Not the Fibrillar Form of the Yeast Prion Ure2p In Vitro
Source: PLoS One. 2015 Jun 26;10(6):e0131789. doi: 10.1371/journal.pone.0131789 (PMC4482727; doi:10.1371/journal.pone.0131789)
Supplement: S1 Table — The position and sequence of each peptide within the primary sequence of Ure2p are indicated, as well as the mass to charge ratio of the peptide that has been fragmented during the nanoLC-MS/MS analysis, the experimental mass (M exp), the theoretical mass (M theor), the mass deviation between the experimental and the theoretical mass (ΔM in ppm) and the mascot ion score for the MS/MS match (score). (PDF) [file pone.0131789.s001.pdf]

**S1 Table: List of peptides generated upon soluble Ure2p degradation by the 26S proteasome after 1, 2 or 3 hours of incubation and identified by nanoLC-LTQ-Orbitrap mass spectrometry.** The position and sequence of each peptide within the primary sequence of Ure2p are indicated, as well as the mass to charge ratio of the peptide that has been fragmented during the nanoLC-MS/MS analysis, the experimental mass (M exp), the theoretical mass (M theor), the mass deviation between the experimental and the theoretical mass (ΔM in ppm) and the mascot ion score for the MS/MS match (score).

| soluble Ure2p + 26S / t 1 h | | | | | | | |
|---|---|---|---|---|---|---|---|
| Start | End | Peptide sequence | m/z | M exp | M theor | ΔM (ppm) | score |
| 1 | 12 | MMNNNGNQVSNL | 668,2929 | 1334,5713 | 1334,5707 | 0,43 | 57 |
| 1 | 13 | MMNNNGNQVSNLS | 711,8073 | 1421,6001 | 1421,6027 | -1,87 | 51 |
| 1 | 14 | MMNNNGNQVSNLSN | 768,83 | 1535,6454 | 1535,6456 | -0,18 | 52 |
| 1 | 15 | MMNNNGNQVSNLSNA | 804,3486 | 1606,6827 | 1606,6828 | -0,029 | 48 |
| 1 | 16 | MMNNNGNQVSNLSNAL | 860,8917 | 1719,7689 | 1719,7668 | 1,21 | 43 |
| 1 | 17 | MMNNNGNQVSNLSNALR | 946,9383 | 1891,862 | 1891,8628 | -0,43 | 50 |
| 2 | 12 | MNNNGNQVSNL | 610,7698 | 1219,525 | 1219,5251 | -0,1 | 53 |
| 2 | 14 | MNNNGNQVSNLSN | 703,3101 | 1404,6056 | 1404,6052 | 0,29 | 65 |
| 2 | 15 | MNNNGNQVSNLSNA | 738,8284 | 1475,6422 | 1475,6423 | -0,058 | 42 |
| 3 | 12 | NNNGNQVSNL | 537,2521 | 1072,4896 | 1072,4897 | -0,13 | 32 |
| 3 | 16 | NNNGNQVSNLSNAL | 729,8504 | 1457,6862 | 1457,6859 | 0,27 | 50 |
| 3 | 17 | NNNGNQVSNLSNALR | 807,9005 | 1613,7865 | 1613,787 | -0,3 | 63 |
| 4 | 16 | NNGNQVSNLSNAL | 672,8285 | 1343,6425 | 1343,6429 | -0,28 | 30 |
| 5 | 16 | NGNQVSNLSNAL | 615,807 | 1229,5995 | 1229,6 | -0,44 | 35 |
| 5 | 17 | NGNQVSNLSNALR | 693,8576 | 1385,7006 | 1385,7011 | -0,33 | 47 |
| 6 | 16 | GNQVSNLSNAL | 558,7854 | 1115,5562 | 1115,5571 | -0,75 | 43 |
| 6 | 17 | GNQVSNLSNALR | 636,8367 | 1271,6589 | 1271,6582 | 0,57 | 26 |
| 10 | 17 | SNLSNALR | 437,7406 | 873,4666 | 873,4668 | -0,17 | 49 |
| 10 | 18 | SNLSNALRQ | 501,77 | 1001,5254 | 1001,5254 | 0,053 | 41 |
| 10 | 19 | SNLSNALRQV | 551,3039 | 1100,5933 | 1100,5938 | -0,41 | 70 |
| 11 | 19 | NLSNALRQV | 507,788 | 1013,5615 | 1013,5618 | -0,28 | 38 |
| 12 | 19 | LSNALRQV | 450,7667 | 899,5188 | 899,5188 | -0,086 | 48 |
| 13 | 21 | SNALRQVNI | 507,7881 | 1013,5616 | 1013,5618 | -0,2 | 40 |
| 13 | 23 | SNALRQVNIGN | 593,3202 | 1184,6259 | 1184,6262 | -0,18 | 39 |
| 13 | 24 | SNALRQVNIGNR | 447,9163 | 1340,7271 | 1340,7273 | -0,098 | 36 |
| 13 | 25 | SNALRQVNIGNRN | 728,3918 | 1454,769 | 1454,7702 | -0,81 | 54 |
| 13 | 26 | SNALRQVNIGNRNS | 514,9415 | 1541,8026 | 1541,8022 | 0,24 | 28 |
| 13 | 27 | SNALRQVNIGNRNSN | 552,9555 | 1655,8447 | 1655,8451 | -0,28 | 41 |
| 13 | 29 | SNALRQVNIGNRNSNTT | 620,32 | 1857,9382 | 1857,9405 | -1,24 | 31 |
| 13 | 31 | SNALRQVNIGNRNSNTTTD | 692,3455 | 2074,0147 | 2074,0151 | -0,2 | 27 |
| 13 | 32 | SNALRQVNIGNRNSNTTTDQ | 735,0314 | 2202,0723 | 2202,0737 | -0,65 | 29 |

| | | | | | | | |
|---|---|---|---|---|---|---|---|
| 15 | 21 | ALRQVNI | 407,2501 | 812,4857 | 812,4868 | -1,39 | 27 |
| 18 | 31 | QVNIGNRNSNTTTD | 767,3661 | 1532,7177 | 1532,7179 | -0,11 | 37 |
| 18 | 32 | QVNIGNRNSNTTTDQ | 831,3951 | 1660,7756 | 1660,7765 | -0,53 | 61 |
| 18 | 33 | QVNIGNRNSNTTTDQS | 874,9111 | 1747,8077 | 1747,8085 | -0,46 | 56 |
| 20 | 31 | NIGNRNSNTTTD | 653,8024 | 1305,5902 | 1305,5909 | -0,55 | 31 |
| 20 | 32 | NIGNRNSNTTTDQ | 717,8319 | 1433,6491 | 1433,6495 | -0,24 | 57 |
| 20 | 35 | NIGNRNSNTTTDQSNI | 874,9111 | 1747,8076 | 1747,8085 | -0,52 | 58 |
| 22 | 37 | GNRNSNTTTDQSNINF | 891,9037 | 1781,7929 | 1781,7929 | 0,048 | 62 |
| 25 | 37 | NSNTTTDQSNINF | 728,3208 | 1454,627 | 1454,6274 | -0,22 | 38 |
| 25 | 38 | NSNTTTDQSNINFE | 792,8422 | 1583,6698 | 1583,67 | -0,12 | 36 |
| 26 | 37 | SNTTTDQSNINF | 671,2994 | 1340,5843 | 1340,5844 | -0,085 | 52 |
| 26 | 38 | SNTTTDQSNINFE | 735,8206 | 1469,6267 | 1469,627 | -0,23 | 42 |
| 27 | 38 | NTTTDQSNINFE | 692,3048 | 1382,5949 | 1382,595 | -0,038 | 37 |
| 28 | 37 | TTTDQSNINF | 570,7619 | 1139,5092 | 1139,5095 | -0,21 | 44 |
| 28 | 38 | TTTDQSNINFE | 635,2832 | 1268,5518 | 1268,5521 | -0,18 | 35 |
| 38 | 50 | EFSTGVNNNNNNN | 719,3023 | 1436,5901 | 1436,5916 | -1,09 | 29 |
| 38 | 54 | EFSTGVNNNNNNNSSSN | 906,8721 | 1811,7296 | 1811,7306 | -0,58 | 43 |
| 38 | 61 | EFSTGVNNNNNNNSSSNNNNVQNN | 870,6981 | 2609,0724 | 2609,0723 | 0,039 | 32 |
| 38 | 62 | EFSTGVNNNNNNNSSSNNNNVQNNN | 908,7117 | 2723,1132 | 2723,1152 | -0,73 | 26 |
| 39 | 61 | FSTGVNNNNNNNSSSNNNNVQNN | 827,6832 | 2480,0277 | 2480,0297 | -0,8 | 38 |
| 71 | 81 | NDNENNIKNTL | 644,8104 | 1287,6063 | 1287,6055 | 0,64 | 32 |
| 73 | 81 | NENNIKNTL | 530,2749 | 1058,5352 | 1058,5356 | -0,33 | 39 |
| 73 | 82 | NENNIKNTLE | 594,7961 | 1187,5777 | 1187,5782 | -0,39 | 51 |
| 73 | 83 | NENNIKNTLEQ | 658,8254 | 1315,6363 | 1315,6368 | -0,33 | 47 |
| 73 | 84 | NENNIKNTLEQH | 485,2395 | 1452,6966 | 1452,6957 | 0,62 | 41 |
| 95 | 103 | SHVEYSRIT | 546,2776 | 1090,5406 | 1090,5407 | -0,075 | 25 |
| 97 | 103 | VEYSRIT | 434,2321 | 866,4496 | 866,4498 | -0,23 | 26 |

**soluble Ure2p + 26S / t 2 h**

| Start | End | Peptide sequence | m/z | M exp | M theor | ΔM (ppm) | score |
|---|---|---|---|---|---|---|---|
| 1 | 12 | MMNNNGNQVSNL | 668,2926 | 1334,5706 | 1334,5707 | -0,034 | 61 |
| 1 | 13 | MMNNNGNQVSNLS | 711,8084 | 1421,6023 | 1421,6027 | -0,32 | 57 |
| 1 | 14 | MMNNNGNQVSNLSN | 768,8299 | 1535,6452 | 1535,6456 | -0,27 | 42 |
| 1 | 15 | MMNNNGNQVSNLSNA | 804,3484 | 1606,6823 | 1606,6828 | -0,25 | 43 |
| 1 | 15 | MMNNNGNQVSNLSNA | 812,3463 | 1622,6779 | 1622,6777 | 0,17 | 57 |
| 1 | 16 | MMNNNGNQVSNLSNAL | 868,8879 | 1735,7612 | 1735,7617 | -0,3 | 65 |
| 1 | 17 | MMNNNGNQVSNLSNALR | 946,9382 | 1891,8619 | 1891,8628 | -0,49 | 77 |
| 2 | 12 | MNNNGNQVSNL | 602,7724 | 1203,5302 | 1203,5302 | 0,03 | 58 |
| 2 | 14 | MNNNGNQVSNLSN | 703,3093 | 1404,604 | 1404,6052 | -0,85 | 43 |
| 2 | 15 | MNNNGNQVSNLSNA | 738,8283 | 1475,6421 | 1475,6423 | -0,14 | 59 |
| 2 | 16 | MNNNGNQVSNLSNAL | 795,3697 | 1588,7248 | 1588,7263 | -0,95 | 53 |
| 2 | 17 | MNNNGNQVSNLSNALR | 881,4187 | 1760,8228 | 1760,8224 | 0,28 | 75 |
| 3 | 12 | NNNGNQVSNL | 537,2521 | 1072,4897 | 1072,4897 | -0,0028 | 30 |
| 3 | 15 | NNNGNQVSNLSNA | 673,3079 | 1344,6013 | 1344,6018 | -0,37 | 39 |
| 3 | 16 | NNNGNQVSNLSNAL | 729,8499 | 1457,6853 | 1457,6859 | -0,4 | 50 |

| | | | | | | | |
|---|---|---|---|---|---|---|---|
| 3 | 17 | NNNGNQVSNLSNALR | 807,9003 | 1613,786 | 1613,787 | -0,6 | 94 |
| 4 | 16 | NNGNQVSNLSNAL | 672,8292 | 1343,6439 | 1343,6429 | 0,71 | 44 |
| 4 | 17 | NNGNQVSNLSNALR | 750,8787 | 1499,7429 | 1499,744 | -0,76 | 75 |
| 6 | 16 | GNQVSNLSNAL | 558,7857 | 1115,5569 | 1115,5571 | -0,19 | 54 |
| 6 | 17 | GNQVSNLSNALR | 636,8365 | 1271,6584 | 1271,6582 | 0,19 | 52 |
| 8 | 17 | QVSNLSNALR | 551,3045 | 1100,5944 | 1100,5938 | 0,59 | 43 |
| 10 | 17 | SNLSNALR | 437,7406 | 873,4666 | 873,4668 | -0,24 | 56 |
| 10 | 18 | SNLSNALRQ | 501,77 | 1001,5254 | 1001,5254 | 0,053 | 41 |
| 10 | 19 | SNLSNALRQV | 551,3041 | 1100,5936 | 1100,5938 | -0,17 | 64 |
| 11 | 19 | NLSNALRQV | 507,7879 | 1013,5612 | 1013,5618 | -0,57 | 43 |
| 12 | 19 | LSNALRQV | 450,7665 | 899,5185 | 899,5188 | -0,35 | 46 |
| 13 | 21 | SNALRQVNI | 507,7881 | 1013,5617 | 1013,5618 | -0,079 | 40 |
| 13 | 23 | SNALRQVNIGN | 593,3204 | 1184,6263 | 1184,6262 | 0,12 | 45 |
| 13 | 24 | SNALRQVNIGNR | 671,3707 | 1340,7268 | 1340,7273 | -0,36 | 44 |
| 13 | 25 | SNALRQVNIGNRN | 728,392 | 1454,7695 | 1454,7702 | -0,47 | 48 |
| 13 | 27 | SNALRQVNIGNRNSN | 552,9556 | 1655,8449 | 1655,8451 | -0,16 | 58 |
| 13 | 28 | SNALRQVNIGNRNSNT | 586,6379 | 1756,892 | 1756,8928 | -0,47 | 28 |
| 13 | 29 | SNALRQVNIGNRNSNTT | 929,9778 | 1857,941 | 1857,9405 | 0,27 | 32 |
| 13 | 31 | SNALRQVNIGNRNSNTTTD | 692,3459 | 2074,0158 | 2074,0151 | 0,34 | 43 |
| 13 | 32 | SNALRQVNIGNRNSNTTTDQ | 735,0313 | 2202,0721 | 2202,0737 | -0,73 | 45 |
| 15 | 21 | ALRQVNI | 407,2505 | 812,4865 | 812,4868 | -0,33 | 27 |
| 16 | 31 | LRQVNIGNRNSNTTTD | 601,6416 | 1801,903 | 1801,9031 | -0,058 | 33 |
| 16 | 32 | LRQVNIGNRNSNTTTDQ | 644,3274 | 1929,9603 | 1929,9617 | -0,68 | 40 |
| 18 | 31 | QVNIGNRNSNTTTD | 767,3658 | 1532,7171 | 1532,7179 | -0,51 | 47 |
| 18 | 32 | QVNIGNRNSNTTTDQ | 831,3949 | 1660,7752 | 1660,7765 | -0,75 | 64 |
| 18 | 33 | QVNIGNRNSNTTTDQS | 874,9111 | 1747,8076 | 1747,8085 | -0,53 | 54 |
| 20 | 31 | NIGNRNSNTTTD | 653,8029 | 1305,5912 | 1305,5909 | 0,2 | 28 |
| 20 | 32 | NIGNRNSNTTTDQ | 717,8319 | 1433,6493 | 1433,6495 | -0,15 | 27 |
| 20 | 35 | NIGNRNSNTTTDQSNI | 874,9109 | 1747,8072 | 1747,8085 | -0,73 | 49 |
| 20 | 36 | NIGNRNSNTTTDQSNIN | 931,9327 | 1861,8509 | 1861,8514 | -0,27 | 35 |
| 20 | 37 | NIGNRNSNTTTDQSNINF | 1005,4679 | 2008,9212 | 2008,9198 | 0,7 | 56 |
| 20 | 38 | NIGNRNSNTTTDQSNINFE | 1069,9877 | 2137,9608 | 2137,9624 | -0,77 | 30 |
| 22 | 37 | GNRNSNTTTDQSNINF | 891,9037 | 1781,7928 | 1781,7929 | -0,019 | 69 |
| 24 | 37 | RNSNTTTDQSNINF | 806,3713 | 1610,7281 | 1610,7285 | -0,21 | 47 |
| 25 | 37 | NSNTTTDQSNINF | 728,3209 | 1454,6273 | 1454,6274 | -0,053 | 46 |
| 25 | 38 | NSNTTTDQSNINFE | 792,842 | 1583,6695 | 1583,67 | -0,27 | 66 |
| 26 | 37 | SNTTTDQSNINF | 671,2994 | 1340,5842 | 1340,5844 | -0,17 | 55 |
| 26 | 38 | SNTTTDQSNINFE | 735,8206 | 1469,6267 | 1469,627 | -0,23 | 55 |
| 27 | 37 | NTTTDQSNINF | 627,7833 | 1253,5521 | 1253,5524 | -0,26 | 34 |
| 27 | 38 | NTTTDQSNINFE | 692,3044 | 1382,5942 | 1382,595 | -0,57 | 38 |
| 28 | 37 | TTTDQSNINF | 570,7617 | 1139,5089 | 1139,5095 | -0,53 | 41 |
| 28 | 38 | TTTDQSNINFE | 635,2835 | 1268,5525 | 1268,5521 | 0,31 | 32 |
| 29 | 37 | TTDQSNINF | 520,238 | 1038,4615 | 1038,4618 | -0,27 | 36 |

| | | | | | | | |
|---|---|---|---|---|---|---|---|
| 30 | 38 | TDQSNINFE | 534,236 | 1066,4574 | 1066,4567 | 0,62 | 26 |
| 32 | 39 | QSNINFEF | 499,7326 | 997,4506 | 997,4505 | 0,15 | 29 |
| 38 | 52 | EFSTGVNNNNNNNSS | 806,3348 | 1610,655 | 1610,6557 | -0,42 | 27 |
| 38 | 54 | EFSTGVNNNNNNNSSSN | 906,8724 | 1811,7302 | 1811,7306 | -0,24 | 60 |
| 38 | 61 | EFSTGVNNNNNNNSSSNNNNVQNN | 870,6977 | 2609,0712 | 2609,0723 | -0,39 | 33 |
| 38 | 62 | EFSTGVNNNNNNNSSSNNNNVQNNN | 908,7125 | 2723,1156 | 2723,1152 | 0,14 | 30 |
| 39 | 61 | FSTGVNNNNNNNSSSNNNNVQNN | 827,6826 | 2480,0259 | 2480,0297 | -1,54 | 42 |
| 43 | 78 | VNNNNNNNSSSNNNNVQNNNSGRNGSQNNDNENNIK | 1315,566 | 3943,6763 | 3943,6758 | 0,14 | 30 |
| 62 | 81 | NSGRNGSQNNDNENNIKNTL | 735,0074 | 2202,0003 | 2202,0009 | -0,27 | 27 |
| 71 | 82 | NDNENNIKNTLE | 709,3321 | 1416,6496 | 1416,6481 | 1,11 | 26 |
| 73 | 81 | NENNIKNTL | 530,2751 | 1058,5356 | 1058,5356 | 0,027 | 32 |
| 73 | 82 | NENNIKNTLE | 594,7961 | 1187,5777 | 1187,5782 | -0,39 | 44 |
| 73 | 83 | NENNIKNTLEQ | 658,8257 | 1315,6369 | 1315,6368 | 0,12 | 58 |
| 73 | 84 | NENNIKNTLEQH | 727,355 | 1452,6954 | 1452,6957 | -0,19 | 57 |
| 73 | 87 | NENNIKNTLEQHRQQ | 622,6446 | 1864,9121 | 1864,9139 | -0,97 | 47 |
| 73 | 89 | NENNIKNTLEQHRQQQQ | 708,0168 | 2121,0287 | 2121,0311 | -1,12 | 30 |
| 95 | 103 | SHVEYSRIT | 546,2774 | 1090,5402 | 1090,5407 | -0,42 | 31 |
| **soluble Ure2p + 26S / t 3 h** | | | | | | | |
| **Start** | **End** | **Peptide sequence** | **m/z** | **M exp** | **M theor** | **ΔM (ppm)** | **score** |
| 1 | 12 | MMNNNGNQVSNL | 668,2924 | 1334,5703 | 1334,5707 | -0,3 | 65 |
| 1 | 13 | MMNNNGNQVSNLS | 711,8083 | 1421,602 | 1421,6027 | -0,49 | 54 |
| 1 | 14 | MMNNNGNQVSNLSN | 768,8297 | 1535,6447 | 1535,6456 | -0,58 | 55 |
| 1 | 15 | MMNNNGNQVSNLSNA | 804,3483 | 1606,6821 | 1606,6828 | -0,4 | 59 |
| 1 | 16 | MMNNNGNQVSNLSNAL | 860,891 | 1719,7674 | 1719,7668 | 0,36 | 68 |
| 1 | 17 | MMNNNGNQVSNLSNALR | 946,9387 | 1891,8628 | 1891,8628 | -0,038 | 72 |
| 2 | 12 | MNNNGNQVSNL | 610,7697 | 1219,5249 | 1219,5251 | -0,22 | 52 |
| 2 | 13 | MNNNGNQVSNLS | 646,2884 | 1290,5622 | 1290,5622 | -0,01 | 41 |
| 2 | 14 | MNNNGNQVSNLSN | 703,3098 | 1404,6051 | 1404,6052 | -0,068 | 41 |
| 2 | 15 | MNNNGNQVSNLSNA | 738,8287 | 1475,6429 | 1475,6423 | 0,44 | 45 |
| 2 | 16 | MNNNGNQVSNLSNAL | 795,3709 | 1588,7273 | 1588,7263 | 0,59 | 33 |
| 2 | 17 | MNNNGNQVSNLSNALR | 881,4177 | 1760,8208 | 1760,8224 | -0,9 | 53 |
| 3 | 12 | NNNGNQVSNL | 537,252 | 1072,4895 | 1072,4897 | -0,25 | 34 |
| 3 | 15 | NNNGNQVSNLSNA | 673,3075 | 1344,6004 | 1344,6018 | -1,01 | 62 |
| 3 | 16 | NNNGNQVSNLSNAL | 729,8502 | 1457,6858 | 1457,6859 | -0,058 | 49 |
| 3 | 17 | NNNGNQVSNLSNALR | 807,9006 | 1613,7867 | 1613,787 | -0,15 | 104 |
| 4 | 12 | NNGNQVSNL | 480,2306 | 958,4466 | 958,4468 | -0,25 | 32 |
| 4 | 16 | NNGNQVSNLSNAL | 672,8286 | 1343,6427 | 1343,6429 | -0,19 | 34 |
| 4 | 17 | NNGNQVSNLSNALR | 750,8794 | 1499,7442 | 1499,744 | 0,13 | 88 |
| 5 | 16 | NGNQVSNLSNAL | 615,807 | 1229,5995 | 1229,6 | -0,44 | 45 |
| 5 | 17 | NGNQVSNLSNALR | 693,8575 | 1385,7005 | 1385,7011 | -0,42 | 64 |
| 6 | 16 | GNQVSNLSNAL | 558,7856 | 1115,5567 | 1115,5571 | -0,32 | 59 |
| 6 | 17 | GNQVSNLSNALR | 636,8359 | 1271,6573 | 1271,6582 | -0,68 | 48 |
| 8 | 17 | QVSNLSNALR | 551,304 | 1100,5935 | 1100,5938 | -0,28 | 44 |
| 10 | 17 | SNLSNALR | 437,7405 | 873,4665 | 873,4668 | -0,38 | 46 |

| | | | | | | | |
|---|---|---|---|---|---|---|---|
| 10 | 18 | SNLSNALRQ | 501,77 | 1001,5255 | 1001,5254 | 0,11 | 45 |
| 10 | 19 | SNLSNALRQV | 551,304 | 1100,5935 | 1100,5938 | -0,28 | 76 |
| 11 | 19 | NLSNALRQV | 507,7881 | 1013,5616 | 1013,5618 | -0,2 | 46 |
| 12 | 19 | LSNALRQV | 450,7668 | 899,519 | 899,5188 | 0,18 | 45 |
| 13 | 21 | SNALRQVNI | 507,7881 | 1013,5617 | 1013,5618 | -0,02 | 40 |
| 13 | 22 | SNALRQVNIG | 536,2988 | 1070,5831 | 1070,5832 | -0,12 | 30 |
| 13 | 23 | SNALRQVNIGN | 593,3204 | 1184,6262 | 1184,6262 | 0,024 | 45 |
| 13 | 24 | SNALRQVNIGNR | 671,3709 | 1340,7273 | 1340,7273 | 0,003 | 70 |
| 13 | 25 | SNALRQVNIGNRN | 485,9304 | 1454,7694 | 1454,7702 | -0,52 | 47 |
| 13 | 26 | SNALRQVNIGNRNS | 514,9413 | 1541,8022 | 1541,8022 | -0,016 | 35 |
| 13 | 27 | SNALRQVNIGNRNSN | 552,9556 | 1655,8451 | 1655,8451 | -0,047 | 57 |
| 13 | 28 | SNALRQVNIGNRNSNT | 586,6379 | 1756,8918 | 1756,8928 | -0,57 | 28 |
| 13 | 29 | SNALRQVNIGNRNSNTT | 620,3203 | 1857,9391 | 1857,9405 | -0,76 | 53 |
| 13 | 31 | SNALRQVNIGNRNSNTTTD | 692,3453 | 2074,0142 | 2074,0151 | -0,46 | 45 |
| 13 | 32 | SNALRQVNIGNRNSNTTTDQ | 735,0314 | 2202,0723 | 2202,0737 | -0,65 | 44 |
| 13 | 33 | SNALRQVNIGNRNSNTTTDQS | 764,0419 | 2289,1038 | 2289,1057 | -0,86 | 29 |
| 15 | 25 | ALRQVNIGNRN | 418,9056 | 1253,695 | 1253,6952 | -0,19 | 29 |
| 15 | 27 | ALRQVNIGNRNSN | 485,9307 | 1454,7702 | 1454,7702 | -0,024 | 38 |
| 16 | 32 | LRQVNIGNRNSNTTTDQ | 644,3272 | 1929,9598 | 1929,9617 | -0,96 | 33 |
| 18 | 31 | QVNIGNRNSNTTTD | 767,3658 | 1532,717 | 1532,7179 | -0,59 | 48 |
| 18 | 32 | QVNIGNRNSNTTTDQ | 831,3951 | 1660,7757 | 1660,7765 | -0,46 | 81 |
| 18 | 33 | QVNIGNRNSNTTTDQS | 874,9112 | 1747,8078 | 1747,8085 | -0,39 | 58 |
| 19 | 32 | VNIGNRNSNTTTDQ | 767,3676 | 1532,7205 | 1532,7179 | 1,72 | 42 |
| 20 | 31 | NIGNRNSNTTTD | 653,8025 | 1305,5905 | 1305,5909 | -0,28 | 34 |
| 20 | 32 | NIGNRNSNTTTDQ | 717,8317 | 1433,6488 | 1433,6495 | -0,5 | 66 |
| 20 | 35 | NIGNRNSNTTTDQSNI | 874,9112 | 1747,8079 | 1747,8085 | -0,32 | 49 |
| 20 | 36 | NIGNRNSNTTTDQSNIN | 931,9321 | 1861,8496 | 1861,8514 | -0,99 | 44 |
| 20 | 37 | NIGNRNSNTTTDQSNINF | 1005,467 | 2008,9195 | 2008,9198 | -0,16 | 56 |
| 20 | 38 | NIGNRNSNTTTDQSNINFE | 1069,9877 | 2137,9608 | 2137,9624 | -0,77 | 44 |
| 22 | 37 | GNRNSNTTTDQSNINF | 891,9024 | 1781,7902 | 1781,7929 | -1,47 | 68 |
| 23 | 37 | NRNSNTTTDQSNINF | 863,392 | 1724,7695 | 1724,7714 | -1,09 | 32 |
| 24 | 37 | RNSNTTTDQSNINF | 806,3709 | 1610,7273 | 1610,7285 | -0,75 | 33 |
| 25 | 37 | NSNTTTDQSNINF | 728,321 | 1454,6274 | 1454,6274 | 0,03 | 47 |
| 25 | 38 | NSNTTTDQSNINFE | 792,8422 | 1583,6698 | 1583,67 | -0,12 | 50 |
| 26 | 37 | SNTTTDQSNINF | 671,2992 | 1340,5838 | 1340,5844 | -0,46 | 50 |
| 26 | 38 | SNTTTDQSNINFE | 735,8211 | 1469,6277 | 1469,627 | 0,43 | 59 |
| 27 | 38 | NTTTDQSNINFE | 692,3049 | 1382,5953 | 1382,595 | 0,22 | 30 |
| 28 | 37 | TTTDQSNINF | 570,7619 | 1139,5092 | 1139,5095 | -0,21 | 38 |
| 28 | 38 | TTTDQSNINFE | 635,2835 | 1268,5525 | 1268,5521 | 0,31 | 38 |
| 29 | 37 | TTDQSNINF | 520,238 | 1038,4615 | 1038,4618 | -0,27 | 28 |
| 30 | 37 | TDQSNINF | 469,7144 | 937,4142 | 937,4141 | 0,047 | 30 |
| 30 | 39 | TDQSNINFEF | 607,7696 | 1213,5246 | 1213,5251 | -0,41 | 25 |
| 32 | 39 | QSNINFEF | 499,7327 | 997,4508 | 997,4505 | 0,29 | 26 |
| 38 | 49 | EFSTGVNNNNNN | 662,282 | 1322,5494 | 1322,5487 | 0,53 | 52 |
| 38 | 50 | EFSTGVNNNNNNN | 719,3024 | 1436,5902 | 1436,5916 | -1 | 45 |

| | | | | | | | |
|---|---|---|---|---|---|---|---|
| 38 | 52 | EFSTGVNNNNNNNSS | 806,3352 | 1610,6558 | 1610,6557 | 0,1 | 28 |
| 38 | 54 | EFSTGVNNNNNNNSSSN | 906,8721 | 1811,7296 | 1811,7306 | -0,58 | 69 |
| 38 | 56 | EFSTGVNNNNNNNSSSNNN | 1020,9155 | 2039,8165 | 2039,8165 | 0,012 | 28 |
| 38 | 62 | EFSTGVNNNNNNNSSSNNNNVQNNN | 908,712 | 2723,1141 | 2723,1152 | -0,4 | 51 |
| 39 | 61 | FSTGVNNNNNNNSSSNNNNVQNN | 827,6838 | 2480,0295 | 2480,0297 | -0,061 | 36 |
| 55 | 77 | NNNVQNNNSGRNGSQNNDNENNI | 848,6982 | 2543,0729 | 2543,0729 | -0,0055 | 25 |
| 62 | 81 | NSGRNGSQNNDNENNIKNTL | 735,0077 | 2202,0012 | 2202,0009 | 0,15 | 34 |
| 71 | 81 | NDNENNIKNTL | 644,8101 | 1287,6057 | 1287,6055 | 0,17 | 36 |
| 71 | 82 | NDNENNIKNTLE | 709,331 | 1416,6474 | 1416,6481 | -0,44 | 35 |
| 73 | 81 | NENNIKNTL | 530,275 | 1058,5355 | 1058,5356 | -0,1 | 34 |
| 73 | 82 | NENNIKNTLE | 594,7961 | 1187,5776 | 1187,5782 | -0,49 | 44 |
| 73 | 83 | NENNIKNTLEQ | 658,8256 | 1315,6366 | 1315,6368 | -0,15 | 52 |
| 73 | 84 | NENNIKNTLEQH | 727,3548 | 1452,695 | 1452,6957 | -0,43 | 51 |
| 73 | 89 | NENNIKNTLEQHRQQQQ | 708,0173 | 2121,0302 | 2121,0311 | -0,44 | 28 |
| 94 | 103 | RSHVEYSRIT | 416,5544 | 1246,6414 | 1246,6418 | -0,33 | 25 |
| 95 | 103 | SHVEYSRIT | 546,2776 | 1090,5406 | 1090,5407 | -0,075 | 42 |
| 97 | 103 | VEYSRIT | 434,2321 | 866,4496 | 866,4498 | -0,23 | 33 |
